# Neoadjuvant Chemoradiotherapy vesus Chemotherapy alone Followed by Surgery for Resectable Stage III Non-Small-Cell Lung Cancer: a Meta-Analysis

**DOI:** 10.1038/srep34388

**Published:** 2016-09-28

**Authors:** Shan xian Guo, Yan Jian, Ying lan Chen, Yun Cai, Qing yuan Zhang, Fang fang Tou

**Affiliations:** 1 Department of Internal Medicine 2, Jiangxi Cancer Hospital, Nanchang, Jiangxi Province, 330029, China; 2 Medical College of Nanchang University, Nanchang, Jiangxi Province, 330006, China

## Abstract

Neoadjuvant Chemotherapy has been used for the stage III of non-small cell lung cancer (NSCLC) and has shown good clinical effects. However, the survival benefits of radiation therapy added in induction regimens remains controversial. We therefore conducted a meta-analysis of the published clinical trials to quantitatively evaluate the benefit of preoperative chemoradiotherapy. After searching the database of Pubmed, CNKI, EMBASE, ESMO, The Cochrane Library databases, The American Society of Clinical Oncology and Clinical Trials.gov. Trials were selected for meta-analysis if they provided an independent assessment of neoadjuvant chemoradiation and neoadjuvant chemotherapy, odds ratio(OR) for tumor downstaging, mediastinal lymph nodes pathological complete response and local control, hazard ratios (HRs) for 5-year survival and progression-free survival were pooled by the stata software version 12.0. Twelve studies involving 2,724 patients were identified, tumor downstaging (p = 0.01), mediastinal lymph nodes pathological complete responses (p = 0.028) and local control (P = 0.002) were achieved, when compared with neoadjuvant chemotherapy. The meta-analysis demonstrated neither 5-year survival nor progression-free-survival benefit in survival from adding radiation. In conclusion, the addition of radiotherapy into chemotherapy was not superior to neoadjuvant chemotherapy. The higher quality of trials need be investigated combining with the histopathological type and genotyping of lung cancer by clinicians.

Lung cancer is the leading cause of cancer-related death in the world and non-small cell lung cancers (NSCLC) comprise more than 75% of all lung cancers. Approximately one-third of patients with non-small cell lung cancer (NSCLC) are diagnosed with locally advanced (stage III) disease^1^. The vast majority of patients with resectable N2 (ipsilateral lymph node involvement), some patients with N3 (contralateral mediastinal and upraclavicular lymph node involvement) NSCLC are offered surgery^2^, but survival remains disappointingly low even after complete resection. As part of a multimodality therapeutic approach, preoperative induction chemotherapy has been shown to eradication of distant micrometastases early and improve the survival as compared to resection alone^3^.

Phase II data from studies suggest that neoadjuvant chemoradiotherapy are active and well tolerated for patients with good performance status^4^. When compared to induction chemotherapy, whether neoadjuvant chemoradiationtherapy would confer a survival benefit had not been clearly demonstrated by inconsistent results of Phase III studies.

Shah and colleagues^5^ reported a meta-analysis comparing neoadjuvant chemoradiation therapy with chemotherapy alone for potentially operable stage IIIA NSCLC and found no benefit to neoadjuvant chemoradiation therapy over chemotherapy alone with respect to overall survival. Limited data including 1 randomized trials^6^ and 1 phase II trial^7^ are statistically integrated and analyzed in the meta-analysis, significant biases inherent in 2 retrospective studies also decreased the power of meta-analysis. After the literature search completion date (December 2010) of the report by Shah *et al.*, new randomized trials displayed a different trend toward survival with chemoradiation were published recently; new clinical trials enrolled a substantial proportion of Stage IIIB patients with a high disease burden by improvement in radiotherapy (RT) technology. We perform a systematic review and meta-analysis again to ascertain whether the addition of preoperative radiotherapy to chemotherapy would improve survival outcome for NSCLC patients with stage III.

## Materials and Methods

### Eligibility criteria for meta-analysis

Randomized, non randomized and retrospective studies containing potentially operable patients with stage III NSCLC receiving induction chemotherapy and induction chemoradiotherapy were eligible for review. The following criteria for eligibility into this meta-analysis were set before collecting the articles. Odds ratio(OR) and confidence intervals (CIs) of the patients could be calculated at specific time intervals after surgery for tumor downstaging, mediastinal lymph nodes pathological complete response and local control, also the hazard ratios (HRs) and confidence intervals (CIs) for 5-year survival and progression-free survival (PFS) in the article. The articles were published in English between January 1990 and October 2015.

### Collection of published studies

PubMed, Embase, the Cochrane Library (Issue 4, 2007) Databases and the American Society of Clinical Oncology (2002–2015) online conference proceedings were searched in October 2015, Clinical Trials.gov (http://clinical trials.gov) were searched to identify ongoing studies. Relevant articles and abstracts were selected and reviewed by two reviewers, and reference lists were searched for additional trials.

**Figure 1:**
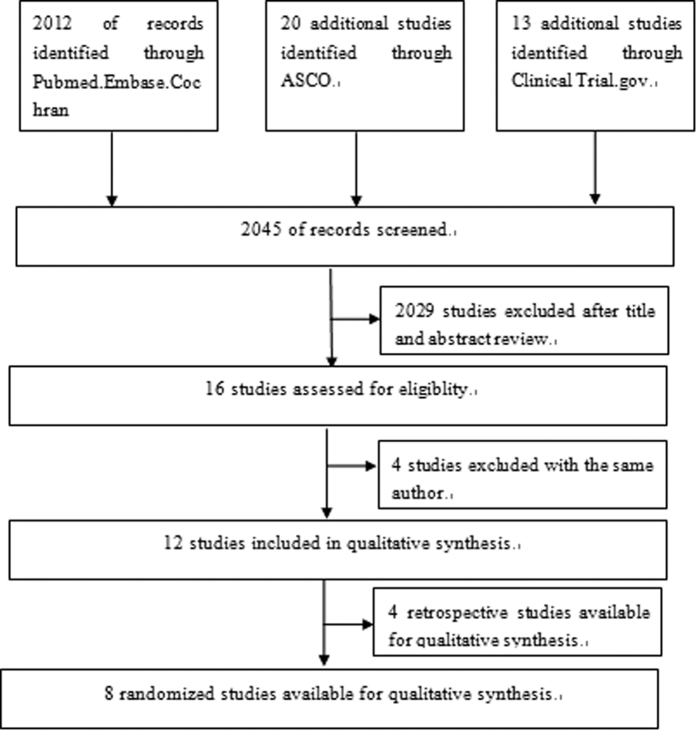
Flow diagram of patients included in systematic review and meta-analysis.

The keywords “non-small cell lung cancer or carcinoma, non-small cell lung or NSCLC” and “induction therapy or chemotherapy or chemoradiotherapy” and “resection or surgery”, hit 2045 citations, the relevant clinical studies were manually selected based on titles and summary analyses. Articles reporting studies unrelated to our question were excluded, and finally only twelve studies (two abstract) were found to fulfill all of our eligibility criteria (Fig. 1).

### Statistical analysis

The Stata software version 12.0 (Stata Corporation, College Station, TX, USA) was used to carry out the meta-analysis. HR and OR with 95% CI was used to combine the data. When these statistical variables were not provided, they were calculated from available numerical data or Kaplan–Meier survival curve^8^. This assumption was tested by performing Chi-squared Q-tests for heterogeneity. A P-value greater than 0.05 for the Q-test indicated lack of heterogeneity among studies, so the fixed-effects model was used for meta-analysis. Otherwise, Dersimonian –Laird random-effect method was used^9^,^10^. We also quantified the effect of heterogeneity using I^2^ statistic which measured the degree of heterogeneity.I^2^ value ranges from 0% to 100%.

Both Begg’s funnel plot and Egger’s test were performed to assess the publication bias. A sensitivity analysis, in which one study was removed at a time, was performed to evaluate result stability.

## Results

We identified 2045 abstracts of which 16 were further assessed for eligibility by full text, After excluding 4 repeated reports, a total of 12 studies involving 2724 patients served as data sources for the present meta-analysis^6^,^7^,^10^,^11^,^12^,^13^,^14^,^15^,^16^,^17^,^18^,^19^, including 8 randomized control trial, 4 retrospective studies (Table 1). The PRISMA flow Diagram for the selection and inclusion of studies is presented in Fig. 1. The main characteristics of the eligible publications are reported in Table 2.

2 studies demonstrated a survival benefit to adding induction radiation to induction chemotherapy versus induction chemotherapy alone, 7 studies did not support it, and 3 studies had no data. In the aspect of disease-free survival, 3 studies indicated that induction chemoradiotherapy were superior to induction chemotherapy, 5 studies supported not, and 4 studies have no data. Subgroup analysis was based on study design, 2 published abstracts and 2 retrospective reviews were unable to be incorporated into the meta-analysis due to lack of available data.

**Figure 2:**
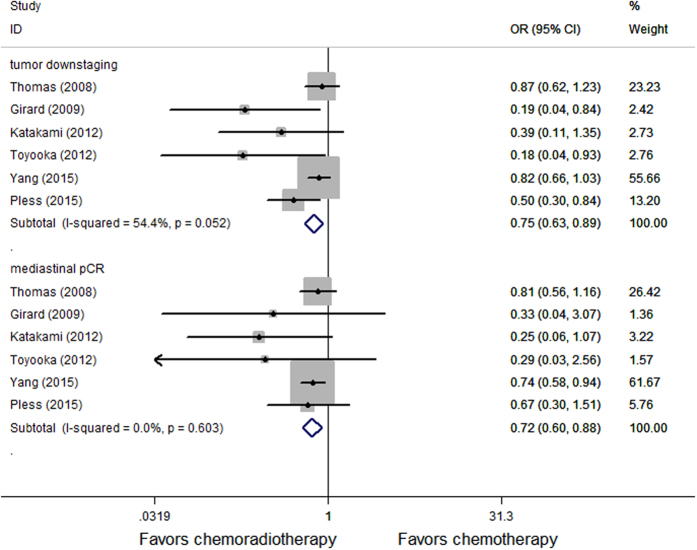
Forest plot of tumor downstaging and mediastinal lymph nodes pathological complete of patients receiving induction chemoradiotherapy versus induction chemotherapy.

**Figure 3:**
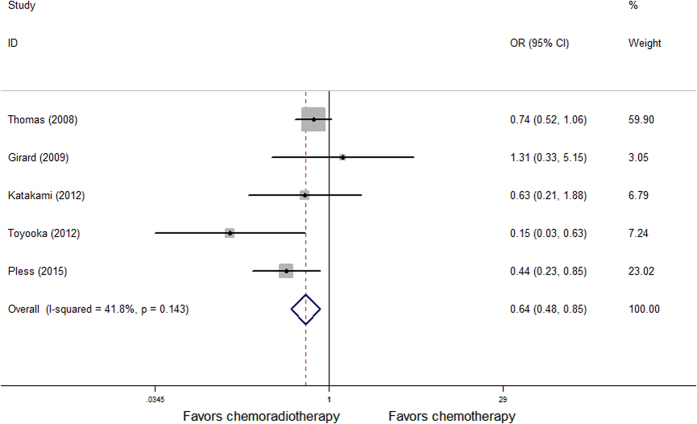
Forest plot of local control of patients receiving induction chemoradiotherapy versus induction chemotherapy.

We can extract the OR and 95%CI through the existing data in 6 randomized controlled trials of 12 studies, the meta-analysis demonstrated induction chemoradiation have benefit in tumor downstaging (OR = 0.75, p = 0.001) and mediastinal lymph nodes pathological complete response (OR = 0.72, p = 0.001) compared with induction chemotherapy (Fig. 2). OR and 95% CI are extracted through the existing data in 5 randomized controlled trials of 12 studies, induction chemoradiation also have benefit in local control (OR = 0.64, p = 0.002) (Fig. 3).

As assessed for hazard ratio of 5-year survival and progression-free survival, the useful data for calculation were obtained directly from the original articles or had to be extrapolated from Kaplan–Meier survival curve. 2 published abstracts and 2 retrospective reviews were unable to be incorporated into the meta-analysis due to lack of available data. Trials by Girard *et al.* and Pless *et al.* were exclude, as Girard *et al.* only provided 3-year survival date and sequential chemo-radiation was administered in trial conducted by Pless *et al.*

**Figure 4:**
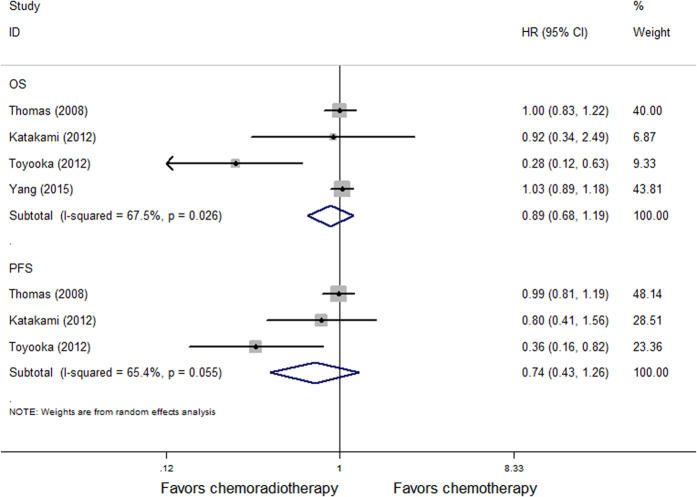
Forest plot of 5-year survival and progression-free survival of patients in randomized studies receiving induction chemoradiotherapy versus induction chemotherapy.

**Figure 5:**
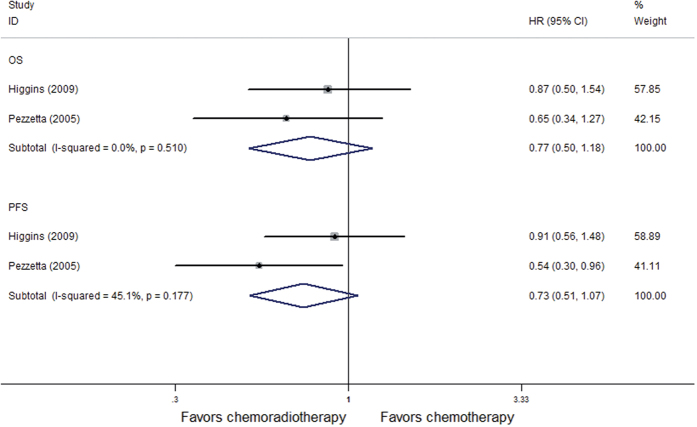
Forest plot of 5-year survival and progression-free survival of patients in retrospective studies receiving induction chemoradiotherapy versus induction chemotherapy.

We conducted a meta analysis of the above 4 randomized controlled trials that administered concurrent chemoradiation using a random effect model. The forest Figure shows no benefit to induction chemoradiotherapy versus induction chemotherapy alone in 5-year OS (HR = 0.89, P = 0.44) nor in PFS (HR = 0.74, P = 0.26) (Fig. 4); Using a fixed effects model for 2 retrospective reviews (I^2^ = 0%), The meta analysis demonstrated no statistically significant benefit to the addition of radiation to induction chemotherapy versus induction chemotherapy alone in OS (HR = 0.77, P = 0.24) nor PFS (HR = 0.73, P = 0.20) (Fig. 5).

There was no indication of publication bias from either Egger’s or Begg’s tests in 5-year survival (Begg p = 0.308; Egger p = 0.267) nor progression-free survival (Begg p = 0.296; Egger p = 0.331). A sensitivity analysis, in which one study was removed at a time, was performed to evaluate result stability. The corresponding pooled OR and HRs were not significantly altered, suggesting stability of our result.

## Discussion

Multimodality therapy is preferable in most subsets of patients with stage III lung cancer. This heterogeneous group of patients can be treated with surgery, chemotherapy, radiation, or both^20^. For individuals with good performance scores, neoadjuvant therapy, followed by surgery, was commonly offered treatment strategy.

Neoadjuvant therapy including induction chemoradiotherapy is feasibly and effective in increasing the resectability of the tumor by decreasing its size^21^,^22^, some studies demonstrated that the addition of radiotherapy to induction chemotherapy improve overall survival compared with induction chemotherapy alone in stage III NSCLC patients, however, other studies did not showed a survival benefit, nor does the result of recent meta-analysis support the findings^5^, which limits the strength of recommendations. We attempted to evaluate and synthesize the available data to provide clinicians with summarized evidence-based information to guide them in taking care of patients with stage III disease.

Induction chemoradiotherapy was well tolerated by stage IIIA or IIIB individuals with good performance scores. Most trials included in the meta-analysis have not observed a difference in surgical complications between the two preoperative regimens. The final result in our meta-analysis shows a benefit of neoadjuvant chemoradiation to the patients with stage III non-small-cell lung cancer in local control, tumor downstaging and mediastinal lymph nodes pathological complete response (pCR). However, the addition of radiotherapy into chemotherapy was not superior to neoadjuvant chemotherapy alone in terms of progression-free survival and 5-year survival.

Induction chemoradiotherapy has multiple potential advantages over chemotherapy alone. The published results showed that the addition of radiation to the preoperative regimen increased the local control rate, which is an important aim of induction treatment^23^.

Although complete resection shown to be a major prognostic factor for survival in multiple studies. The high down-staging rate, pathological complete response and the absence of treatment-related death in CRS arm did not translated into a longer PFS and OS of patients with stage III NSCLC who underwent pulmonary resection, numerous limitations of these trials may hinder a fair assessment of the role of radiation therapy in the induction phase of treatment. Several trials were small and clearly underpowered to detect meaningful differences in outcomes. Phase II trials of Higgins^14^ and Pezzetta^13^ had the limitations as any retrospective analysis. Some of the randomized trials utilized different chemotherapy regimens between the two treatment arms, chemotherapy regiment directly affect pre-existing micrometastases and induces high rates of response for potentially resectable disease. The complicated treatment of patients with postoperative recurrence and metastasis may also affect the result.

The addition of radiotherapy may be not worthwhile in order to improve over survival. Evidence available for meta-analysis were not in favour of it. For patients with good performance status and advanced local disease requiring maximal shrinkage to facilitate complete resection, we recommend considering radiotherapy in combination with chemotherapy in the induction phase of treatment. However, the best therapeutic plan should be achieved through the multidisciplinary cooperation of a team specialized in lung cancer.

Future trials are needed to investigate the roles of individualized chemotherapy and surgery in particular cohorts or settings^24^, and it would be ideal utilize an identical chemotherapy regimen in each treatment. The increasing availability of antibody therapy and tyrosine kinase inhibitors for the treatment of lung cancer might offer new options^25^, Future trials will evaluate the role of targeted therapies in this setting to increase systemic control while decreasing hematological toxicity rates of combined treatment.

Unanswered questions remain about definitive chemoradiotherapy, including the optimal chemotherapy agents; dose, duration, density of chemotherapy^26^, radiation fractionation and radiation dose^27^,^28^,^29^,^30^. However, we were not sure tumor cells would be more sensitive to radiotherapy or chemotherapy when diagnosed, especially for squamous cell carcinoma patients^31^, best modality for assessing response in advanced disease utilizing 18F-FDG PET/CT has been testing^32^, studies on identifying markers to predict the response to CRT should be pursued^33^,^34^, moreover, strategies to select patients for the most appropriate therapy according to the molecular profile of individual tumors could contribute to further improvements in treatment outcome. In conclusion, clinicians needed to investigate the higher quality of trials combined with the histopathological type and genotyping of lung cancer, and probe the best method of treatment for resectable Stage III Non-Small-Cell Lung Cancer.

## Additional Information

**How to cite this article**: Guo, S. x. *et al.* Neoadjuvant Chemoradiotherapy vesus Chemotherapy alone Followed by Surgery for Resectable Stage III Non-Small-Cell Lung Cancer: a Meta-Analysis. *Sci. Rep.* **6**, 34388; doi: 10.1038/srep34388 (2016).

## Figures and Tables

**Table 1:** Studies Included in Systematic Review and Meta-Analyses.

First Author	Publish Years	Study Design	Study Years	Number of Patients		Median Survival (months)		3-Year Survival (%)		5-Year Survival (%)	
				Chemo	Chemo RT	Chemo	Chemo RT	Chemo	Chemo RT	Chemo	Chemo RT
Pless *et al.*	2015	Phase III	2001–2012	115	117	26.2	31.7	…	…	…	…
Yang CF *et al.*	2015	RCT	2003–2006	528	834	40.8	39.6	52	55	41	41
Yang H *et al.*	2015	retrospective study	2008–2013	76	8	…	…	…	…	…	…
Toyooka *et al.*	2012	RCT	1995–2010	15	35	…	…	40	74.8	26.7	67.1
Katakami *et al.*	2012	Phase III	2000–2005	28	28	29.9	39.6	39.3	51.7	23.9	37
Girard *et al.*	2010	Phase II	2003–2007	14	32	24.2	12.5 (B)	36	35 (B), 85.8 (C)	…	…
Li *et al.*	2009	retrospective study	1998–2004	62	29	28	30	27.7	35.3	12.4	29.4
Higgins *et al.*	2009	retrospective study	1995–2006	31	70	…	…	39	41	…	…
Thomas *et al.*	2008	RCT	1995–2003	260	264	…	…	26	28	18	21
Pezzetta *et al.*	2005	retrospective study	1994–2003,	36	46	40	105	57	63	…	…
Sauvaget *et al.*	2000	RCT (abstract)	1991–2000	…	…	19	18.5	…	…	…	…
Flecket *et al.*	1993	RCT (abstract)	….	48	48	8.5	17	17	23	…	…

RCT: Randomized control trial. B: armB C: armC.

**Table 2:** Characteristics of Included Studies.

Study	Clinica Stage	Chemo Regimen	Chemo RT Regimen	Mediastinal pCR/Pathological downstaging	PFS	OS
Pless *et al.*	IIIA (N2)	DP	DP + SRT (44 Gy)	…/…	No (p = 0.67)	No (…)
Yang CF *et al.*	IIIA (N2)	…	…CRT (<40Gy, 40–50Gy, 50–60Gy, >60Gy)	Yes (45.4%vs32.5% p < 0.01)/Yes (58%vs46%, P < 0.01)	…	No (p = 0.73)
Yang H *et al.*	IIIA (N2)	…	…	…/…	…	…
Toyooka *et al.*	III (N2/3)	IP	DP + CRT (40–46 Gy)	NR (20.6%vs6.7%)/Yes (45.7%vs13.3% p = 0.021)	Yes (p = 0.015)	Yes (p = 0.002)
Katakami *et al.*	IIIA (N2)	DC	DC + CRT (40G y)	NR (32.1%vs10.7%)/No (40%vs21% p = 0.215)	No (p = 0.187)	No (p = 0.397)
Girard *et al.*	IIIA (N2)	GC (A)	VP (B)/PC (C) + CRT (46Gy)	NR (19%vs7%)/Yes (84%vs57%, p = 0.049)	Yes (p = 0.035)	No (p = 0.268)
Li *et al.*	IIIA (N2)	EP/VP/GP	EP/VP + SRT (40–50 Gy) EP + CRT (40–45 Gy)	…/…	…	…
Higgins *et al.*	III (N2)	VC	VC + CRT (43–60 Gy)	Yes (65%vs35%.p = 0.02)/…	No (p = 0.90)	No (p = 0.65)
Thomas *et al.*	III	EP/VC	EP + CRT (45 Gy)	Yes (60%vs20% p < 0.0001)/Yes (46%vs29% p = 0.02)	No (…)	No (…)
Pezzetta *et al.*	III (N2)	DP	DP + SRT (44 Gy) VP + CRT (44 Gy)	Yes (p < 0.01)/Yes (p < 0.01)	Yes (p = 0.04)	No (p = 0.38)
Sauvaget *et al.*	T4 or N2	MVP	MVP + CRT (40 Gy)	…/Yes (75% vs 55%)	…	No (…)
Flecket *et al.*	T4 or N2	MVP	MVP + CRT (30Gy)	…	…	…

DP: Docetaxel + Cisplati; IP: Irinotecan + Cisplatin; DC: Docetaxel + Carboplatin; GC: Gemcitabine + Cisplatin; VP: Vinorelbine + Cisplatin; VC: Vinorelbine + Carboplatin; PC: Paclitaxel + Carboplatin; EP: Etoposide + Cisplatin; MVP: Mitomycin + Vinblastine + Cisplatin; NR: No Report; SRT: Sequential Radiochemotherapy; CRT: Concomitant Radiochemotherapy.
